# Visual, Non-Destructive, and Destructive Investigations of Polyethylene Pipes with Inhomogeneous Carbon Black Distribution for Assessing Degradation of Structural Integrity

**DOI:** 10.3390/polym14051067

**Published:** 2022-03-07

**Authors:** Taesik Kim, Suleyman Deveci, Inmo Yang, Bob Stakenborghs, Sunwoong Choi

**Affiliations:** 1 Department of Polymer Science and Engineering, Hannam University, Daejeon 34054, Korea; taesik.kim93@gmail.com (T.K.); inmomni@kcl.re.kr (I.Y.); 2 Innovation Centre, Borouge Pte Ltd., Abu Dhabi P.O. Box 6951, United Arab Emirates; suleyman.deveci@borouge.com; 3 Korea Conformity Laboratories, Seoul 08503, Korea; 4 Advanced Microwave Imaging, Baton Rouge, LA 70815, USA; rjstak@advancedmwimaging.com

**Keywords:** carbon black, “windows”, degradation, polyethylene pipe, non-destructive test, destructive test

## Abstract

Carbon black (CB) is used in polyethylene (PE) pipes to protect against thermal and photooxidation. However, when CB is not properly dispersed in the PE matrix during processing, white regions having little or no CB concentration, known as “windows,” appear within the CB/PE mixed black compound. In some cases, windows can drastically affect the structural integrity of both the pipe and butt fusion joint. In this work, PE pipes with varying amounts of windows were investigated for their characteristic window patterns, as well as quantifying the area fraction of windows (% windows). Tensile test on specimens with known % windows determined a critical limit above which the fracture strain rapidly degrades. Micro-tensile and micro-indentation results showed tear initiation at the window–black PE matrix boundary; however, they did not confirm the mechanism of tear initiation. In support of this work, a method of making thin shavings of a whole pipe cross section was developed, and the best viewing windows under cross-polarized monochromatic light were identified. In addition, a phased array ultrasonic test (PAUT) and microwave imaging (MWI) were directly applied to the pipe and confirmed the presence and patterns of the windows.

## 1. Introduction

Polyethylene (PE) pipes have been successfully utilized in a wide range of applications in water, gas, and power utilities for many years. More recently, nuclear power plants also began to use PE pipes for safety class nuclear applications [1]. A successful track record of polyethylene pipeline systems in these demanding applications replacing metallic alternatives lies in its excellent chemical and corrosion resistance and leak-free fusion jointing capabilities.

The short- and long-term mechanical integrity of PE pipeline systems in field use depends on the quality of the PE material used, additives against thermal and photodegradation, and extrusion process, as well as fusion jointing integrity [2]. Furthermore, as most PE pipe systems are laid above ground and exposed to direct sunlight, protection of PE against UV-induced thermal and photodegradation has paramount importance [3].

The use of carbon black (CB) in polyethylene (PE) pipes is known to be the most economically effective measure against exposure to thermal and photodegradation [4,5,6,7]. To gain optimum performance from the carbon black addition, CB particles being utilized at required concentrations need to be well dispersed and distributed among the natural pipe resin matrix, as CB particles are known to dictate the performance of the polyethylene (PE) pipes. For high-molecular-weight PE pipe-grade resins for pressure pipes, the addition of CB with an average particle size of 20 nm to the polymer matrix at 2–2.5 weight percent distribution as per ISO 4427 [8] requirements is a challenging task and requires specialized processing equipment to obtain an adequate level shearing for proper CB dispersion [9].

Much of the work in the past with CB particles in PE pipes was more focused on their interaction with UV and thermal environments to prevent photo- and thermal oxidation [10,11,12,13,14,15,16,17]. The effect of CB on the mechanical behavior of PE pipes has been reported. The CB content [7,17,18], type [10], and dispersion [9,19] in the PE matrix all affected the bulk mechanical properties of PE. The key to the optimum use of CB rests on its proper dispersion and distribution mixing in the PE resin matrix. With regard to the CB particle size [20], using 45 nm or 25 nm did not show a difference in the mechanical properties tested. It was noted that a smaller size would be potentially advantageous for improving UV stability beyond 10 years of weathering.

Recently, brittle fractures on PE pipes occurred during installation pressure testing, and the failure analysis revealed fracture surfaces containing islands of white areas among the dark polyethylene matrix [21]. These white areas are known as “windows” and represent areas of little or no CB presence as a result of insufficient mixing between CB particles and the PE resin [2,22,23] during the extrusion processing. Such a lack of mixing leads to poor distribution and dispersion of CB particles in PE resin and is known to cause windows to appear. Hence, the presence of windows is considered a measure of the degree of processing [24,25]. This can occur when carbon black masterbatch (CBMB) and a non-pigmented PE compound are melt processed together (in-line compounding) during a single screw pipe extrusion, where the screw and mixing elements are not properly designed to provide the necessary dispersion and distribution of CB in the PE material. Therefore, to prevent dependence on the proper single screw design for the pipe extrusion, the use of a ready-made PE compound (pre-compound) is required in the water pipe standard of ISO 4427 [8]. The advantage of using a pre-compound is that good melt homogeneity and mixing during single screw pipe extrusion are relatively simple, as only uniform heating of pellets during a given extrusion condition is needed [20].

PE pipe qualities produced by in-line compounding and pre-compound were compared [20,24,26]. In-line compounding was shown to require a rigorous screw and barrel design [27] to achieve pipe mechanical properties equivalent to pre-compounds (e.g., slow crack growth, rapid crack propagation, hydrostatic burst, and sustained butt fusion strength). In another study [28], in-line compounding was done in an extrusion system designed for pre-compound. The pipe properties (slow crack growth and rapid crack propagation) did not show a real difference from the pre-compound. However, it was mentioned that in-line compounding required CBMB and NPC in the pellet form and their adequate dry blending before the extrusion. Additionally, a careful choice of screw design and the evaluation of in-line mixing with extruder throughput was stressed. In another study, pre-compound and in-line compounding gave similar PENT values; however, data scatter was reported as larger with in-line compound and pre-compound was more reproducible [26].

There exists very limited literature concerning the effect of windows’ presence on the structural integrity of pipes and their butt fusion joints [21,22,23,29,30,31,32,33,34,35]. Rapid crack propagation field failures were noted on in-line compound 315 SDR 11 PE water pipes and butt fusions in non-safety class nuclear power installation [21]. The failure analysis revealed that the PE pipe walls contained windows and the fracture strain decreased by as much as 15 times compared to pre-compound pipes [21]. The fatigue crack growth behavior on the failed pipe was carried out using a stiff constant K specimen [32]. The crack growth rate was shown to be about 10 times faster when the windows were present. In a later study, the slow crack growth in butt fusion joints made from safety class nuclear PE pipes with various degrees of windows did not indicate a real difference due to a large data scatter observed [29]. However, in the following study [22], the data were separately superimposed on the time to failure curve. They essentially indicated the accelerating effect of the slow crack growth with increasing the window amount.

The mechanical and fracture behaviors of windowed pipes and butt fusion joints involving controlled windows’ levels (low, medium, and high levels) were recently reported [22,23,30,31,35]. They indicated the post-neck strains decreasing drastically, and a transition from normally ductile to brittle fracture surface was observed when windows above a certain level were present [22,23,30,31]. It was also reported that tear initiation was observed at the window–black compound boundary at cold drawing strains; such premature interfacial separation was attributed to the observed property reduction. Similarly, for butt fusion joints, elongation [22,31] or energy to break [31,35] decreased with windows. Such a reduction was first shown with a dog-bone tensile specimen (ISO 527 Type IA) [36] removed from a butt fusion area [22] and later with waisted tensile specimens (ISO 13953) [31,35,37]. In both cases, the elongation was significantly reduced with the medium- and high-level windows specimens. With dog-bone tensile specimens, failure occurred after necking, while before-necking failures were observed in waisted tensile specimens.

In terms of observing window patterns and quantifying % windows, most of the work done used microtomed thin films obtained from small sections (e.g., 10 mm × 10 mm) taken from the whole pipe cross section [23,24,29,31,35,38]. The grayscale threshold and pixel count method were used to establish % windows in both in-flow and cross-flow directions for pipes and butt fusion zones [23,31]. They also determined the size of the most prominent widows’ area, and at least a 100-μm size window area was needed to affect the mechanical property [23].

This work investigated methods for observing and quantifying windows in a whole pipe cross section. First, visual methods using white and monochromatic light as well as using polarized light are described. In addition, windows, as viewed by the non-destructive tests (phased array ultrasonic testing and microwave imaging), are presented. Additionally, a destructive test to determine the limiting area fraction of windows (% windows) was demonstrated. Finally, in an attempt to better understand the mechanism of failure initiation in the presence of windows, results from the micro-tensile tests on shavings along with micro-indentation are presented.

## 2. Materials and Methods

### 2.1. Pipe Specimens

The polyethylene (PE) pipe samples of size 110 SDR 5 (110 mm outside diameter (OD), 22 mm pipe wall thickness (t); standard dimensional ratio (SDR) = OD/t) were received as extruded. The pipes were extruded in a production-scale, single screw extrusion line (Reifenhauser, Troisdorf, Germany), having a 60 mm screw diameter, a length-to-diameter ratio (L/D) of 33, and a four-channel spiral die. Four types of PE pipes were produced, and they were distinguished by the level of “windows” present in the pipe wall, as shown in Table 1. Different levels of windows (low, medium, and high) were achieved by utilizing non-pigmented PE compound (NPC) containing antioxidant and carbon black (CB) master-batch (CBMB: 40% CB and 60% carrier resin) in a single screw pipe extruder. CB loading was kept constant for these pipes, and extrusion parameters were maintained the same, except the extrusion throughput was varied to provide different residence times to get various CB distribution characteristics. The reference sample (windows free) was produced in the same extruder using PE powder obtained directly from the polymerization reactor and then compounded with antioxidant and CBMB in a counter-rotating continuous mixer. This reference sample was a pipe produced from a commercial-grade HE3490LS PE compound from Borouge Ltd. (Abu Dhabi, United Arab Emirates).

A detailed description of the compounding, extrusion, melting, and physical properties of the PE pipes utilized in this work is given in a previous publication of one of the authors [23]. CB content, melt flow rate (MFR), and density were similar among all samples (see Table 1), except for the visual windows’ ratings according to ISO 18553 [38]. Thus, sample 1 (windows free reference sample), sample 2 (low windows), sample 3 (medium windows), and sample 4 (high windows) had visual ratings of A1-A2, B-C1, C1, and C1-C2, respectively [23].

### 2.2. Visual Observation of Windows in Whole Pipe Cross Section

#### 2.2.1. Preparation of Whole Pipe Cross Section Shavings

**Figure 1:**
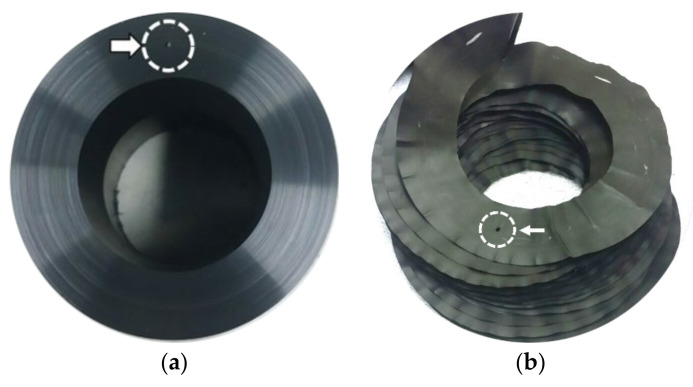
The 110 SDR 5 PE pipe, (**a**) 1 mm locator hole, (**b**) 50 µm thick whole pipe cross-section shavings.

For the visual observation of windows and their patterns in the whole pipe wall cross section, a continuous whole pipe cross-section layer removal method was developed and utilized. This method produced shavings of the whole pipe cross section, as thin as 30 μm thick, on a lathe (Mecca Turn 400 × 750, Namsun, Gwangju, Korea) using high-speed alloy steel (HSS-68 HRC) cutting bit (Figure 1). The shavings were made thin enough to reveal the windows in each pipe. The cutting bit was ground square to a surface roughness of Ra 20 or less, and a major cutting edge of 40 mm long was made to ensure the cutting of the whole pipe wall thickness at all times. The surface roughness of the cutting bit was found to be an important factor as the cutting marks produced on the shavings using higher surface roughness bits tended to obscure the windows, thus making viewing more difficult.

Before cutting in the lathe, a 1 mm diameter hole was drilled into the pipe cross section as a locator hole (Figure 1a) for positioning the shavings in the proper orientation (Figure 1b). In order to make the whole pipe cross-section shavings of various thicknesses, the pipe was turned in the lathe at a speed of 55 rpm with compressed air blowing at the cutting area. This condition minimized the twisting and coiling of the shavings being produced. The cutting speed in the pipe axial direction depended on the thickness of the shaving made.

For shavings greater than 50 µm, ironing at 120 °C, removed twists and coils to flatten the whole pipe cross-section shavings (Figure 1b). It was noted that when the shaving thickness was less than 50 µm, the surface was no longer flat. Even after ironing, many local wrinkles appeared, which distorted the sample and made direct viewing of the windows difficult.

#### 2.2.2. Visual Observation of Windows in Whole Pipe Cross Section

The visual observations of the windows were made by cutting enough shavings to reconstruct a whole pipe cross section (Figure 1b) and then placing the reconstruction under the transmitting white light, monochromatic light, and polarized lights for viewing. It was determined that a good resolution and contrast for viewing were offered by placing the shaving between two polarizer plates (ESM-647, Intech-optic, Goyangsi, Korea) in cross polarization. The monochromatic light was produced by a sodium vapor lamp (GEO-NH, GEOlighting, Anseong, Korea), and a white LED lamp (LED lamp, Cityo, Incheon, Korea) was used. A light diffuser plate was used for all observations.

Based on these results, all subsequent viewing and tensile testing were done on 100 µm shavings, which provided windows’ viewing equivalent to the 50 µm shaving and also made a stable specimen for handling and testing.

#### 2.2.3. Measurement of Relative Window Concentrations in the Shavings

The level of windows in samples 1–4 was measured using 100 µm shavings from each pipe sample. Two visual observations were made. A cross-polarized white light was used in one case, and the other used a cross-polarized monochromatic light. A photo of a shaving was converted into a grayscale image with the rest of the background in red. The RGB code number was confirmed as 55-55-55 for the window–black compound boundary using Photoshop, and the color depth below the code number 55 was judged to be a windows-free black compound. The color distribution of the photo was checked using an open-source program [39], which arranged the color of each pixel by a code number. They were then analyzed to calculate the area fraction of windows (% windows) by dividing the number of pixels above 55 by the total number of pixels in the shaving. The shavings from each of the eight segmented sectors (see Section 2.4.1) and the whole pipe cross sections were subjected to a % windows determination.

### 2.3. Observation of Windows by the Non-Destructive Tests

The observation of the windows directly from the pipe samples was investigated using non-destructive test methods. Both phased array ultrasonic test (PAUT) and microwave imaging (MWI) methods were examined.

#### 2.3.1. PAUT

**Figure 2:**
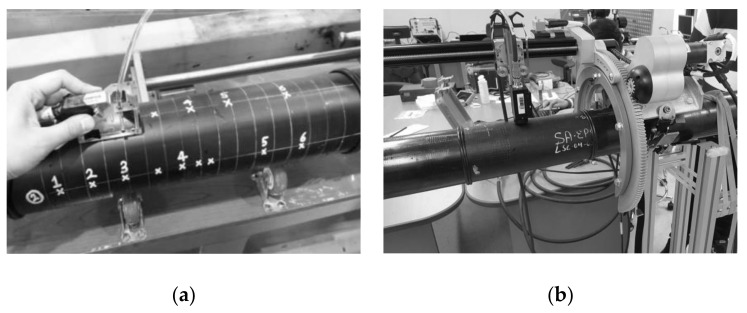
(**a**) PAUT and (**b**) MWI for non-destructive inspection of windows in pipes.

The inspection system for PAUT was an Olympus unit (OmniScan MX2, Tokyo, Japan), consisting of a detector, 2.25-MHz frequency, 32 active elements’ transducer water wedge, encoder, and encoder jig, as shown in Figure 2a. Before the inspection, the pipes were marked with a white marker at regular 2 cm intervals along the inspection length considered. At first, the wedge and the transducer assembly were placed in close contact with the pipe surface, and the pipe was PAUT inspected along the circumferential direction by manually rotating the pipe. This procedure was repeated until the entire area of the pipe was examined. The beam was steered from 0° to +85° by sectorial scanning and focusing the beam at two-thirds of the pipe thickness. After scanning, the merged C-scan images were reproduced from the software (Tomoview 2.10 R25) for viewing the window indications.

#### 2.3.2. MWI

The microwave NDT apparatus used was a single frequency device that operates near 24 GHz (AMWI-SF24G-OW, Advanced Microwave Imaging, Baton Rouge, LA, USA). A microwave sensor consisted of a Gunn diode with a tuned cavity and two Schottky diodes placed in the exiting waveguide section. The microwave sensor was driven in an automated two-axis encoded device that ran axially and circumferentially around the pipe (Figure 2b). An open waveguide acted as the sensor antenna. The Schottky diodes generated a DC voltage in an alternating electric field that was roughly linear with the amplitude and phase of the alternating field. The voltages were recorded at locations along the pipe axial and circumferential scanning path. They were compiled into a viewing algorithm where the voltage variation was assigned a grayscale based on its magnitude, and a complete image of the pipe was created. Because of the very low losses in polyethylene material in the microwave frequency range, the sensor easily captured an image of the entire volume within the sensor aperture range. The open waveguide transmitted a signal that spread out from the aperture opening at a 45-degree angle from the aperture opening; so, the coverage area grew quickly as the beam penetrated the material depth. This ensured beam coverage to the entire pipe cross section; however, it hampered individual resolution of flaws deeper into the part.

### 2.4. Tensile Test

#### 2.4.1. Pipes

**Figure 3:**
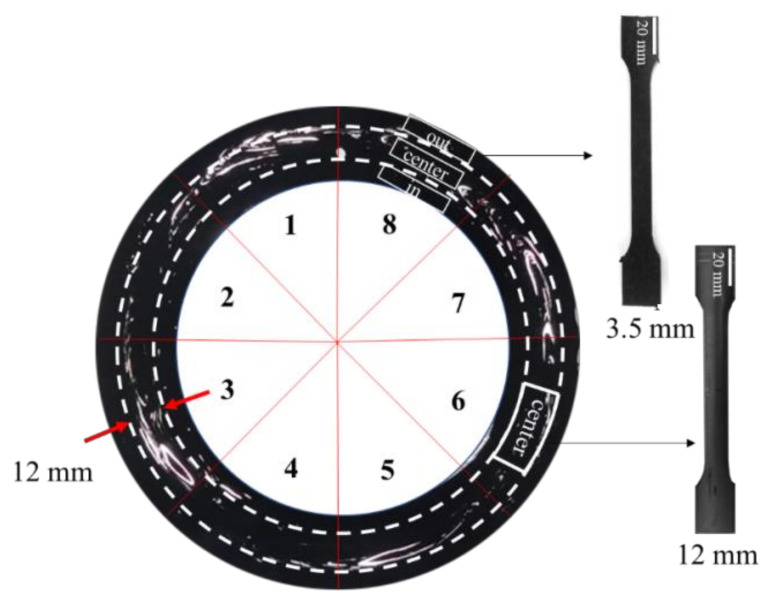
Eight sectors with known % windows where 12 mm thick tensile specimens were taken. The 3.5 mm thick specimen locations are also shown.

Tensile tests on windowed 110 SDR 5 PE pipes were performed using ISO 527-2 [36] Type 1A tensile specimens of 3.5 mm and 12 mm thickness, machined from each pipe sample. Since the windows were observed to reside approximately in the center of the 12 mm thickness portion of the pipe samples, 12 mm specimens were removed from the center of the pipe wall of every eight segmented pipe sectors, as shown in Figure 3. For these eight sectors selected, the % windows were all prior determined. On the other hand, 3.5 mm thick specimens were taken from the outer, central, and inner wall regions (Figure 3) and were used to estimate the change in properties through the pipe wall thickness.

The tensile specimens of 3.5 mm were made first by band-sawing the pipe into eight sectors. Then, a milling machine (Simplex-2, Hwacheon, Gwangju, Korea) was used to obtain the required thickness at their respective positions (Figure 3). The other dimensions were obtained using template machining with a cutter rotating at 3000 rpm. For the 12 mm specimens, the pipes were turned and bored in a lathe (Mecca Turn 400×750, Namsun, Gwangju, Korea) to remove the outer and inner layers until a 12 mm thickness was achieved. The same template machining was used for the final specimen dimensioning.

All specimens were conditioned at 23 °C and 50% RH for at least 24 h before tensile testing. The tensile tests were carried out at conditioning temperature using a universal testing machine (AGS-5, Shimadzu, Kyoto, Japan) equipped with a 50-kN load cell and a dual-camera optical extensometer with up to 800 mm field of view measurement capability. The displacement rates were 100 mm/min and 50 mm/min for 3.5 mm and 12 mm thick specimens, respectively. The gauge length for all specimens was set at 50 mm.

#### 2.4.2. Shavings

**Figure 4:**
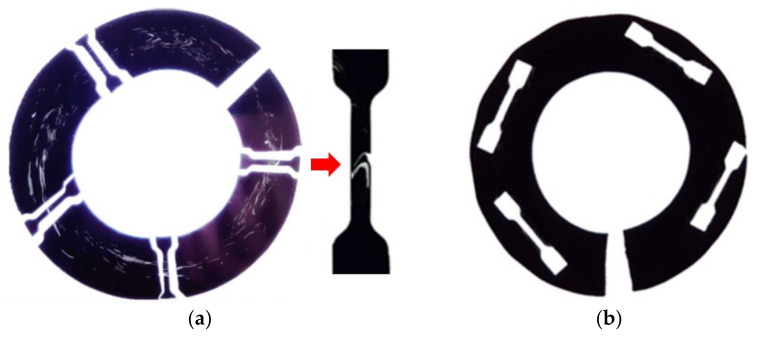
Sampling location of micro-tensile specimens. From (**a**) sample 4 and (**b**) sample 1.

Micro-tensile specimens of ISO 572-2 type 1A [36] on 100 µm thick shavings were made from pipe sample 4 and sample 1, and the photographs are shown in Figure 4. In sample 4, the specimens were punched out perpendicular to the pipe circumference at four high window swirl areas (Figure 4a) and in sample 1, from four positions, as shown in Figure 4b. The specimens were conditioned at 23 °C and 50% RH for at least 24 h prior to the tensile test. Tensile tests were carried out using pneumatic grips on a 500-N load cell at a 100 mm/min cross-head speed (AGS-5, Shimadzu, Kyoto, Japan). No extensometer was used.

### 2.5. Scanning Electron Microscope (SEM)

The fracture surfaces of the failed specimens were observed using a scanning electron microscope (SEM). The specimens were cut, Pt coated in a sputtering machine (AGB7341, Agar Scientific Ltd., Essex, England), and observed under an SEM (JSM -7610F Plus, Jeol Ltd., Tokyo, Japan) using an accelerating potential of 25 kV.

### 2.6. Micro-Indentation

**Figure 5:**
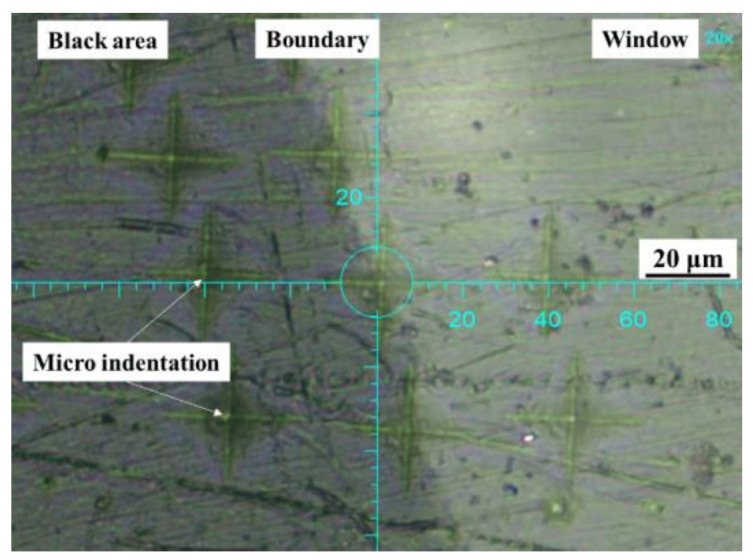
Micro-indentations on black compound, window, and along the boundary.

A micro-indentation test was carried out on a 100 µm shaving using a PICODENTOR HM500 (Helmut Fisher, Sindelfingen, Germany) with a 136° plane angle Vickers indenter that utilized the load-indentation depth method in accordance with ISO 14577-2 [40]. First, a shaving with windows was pressed against the glass slide using a rubber roller to obtain a good physical bond between the two. Once the indentation location was identified (Figure 5), a pre-selected load of 30 mN on a Vickers indenter was applied to the shaving for 6 s for the press-in followed by 5 s of indentation hold time. The load was then removed from the shaving at the same speed as the indenter press-in. A total cycle time of 17 s was used to obtain a load-depth profile from which various mechanical properties were computed using the methods in ISO 14577-1 [41]. Measurements included Martens hardness (HM), indentation hardness (HIT), and modulus of indentation (EIT). Figure 5 shows the micro-indentation positions on the window, the boundary, and the black compound.

## 3. Results and Discussion

**Figure 6:**
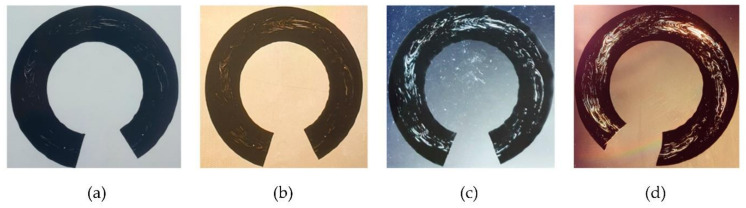
The 100-μm shaving viewed under: (**a**) white, (**b**) monochromatic, (**c**) cross-polarized white, and (**d**) cross-polarized monochromatic lights.

**Figure 7:**
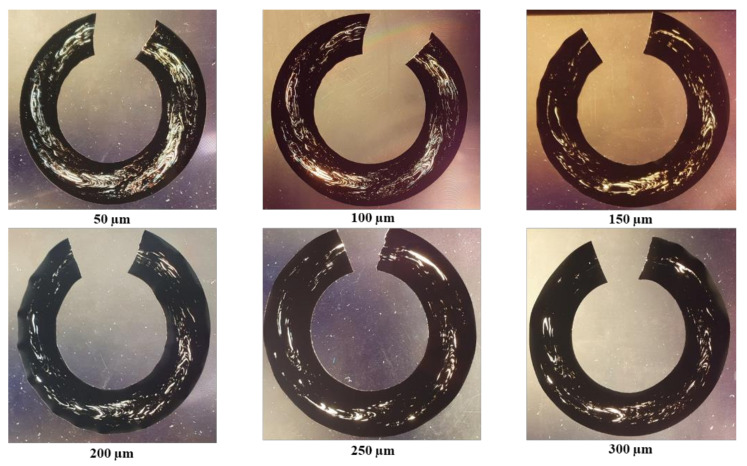
Windows’ observation of the whole pipe cross sections in shavings of different thicknesses under cross-polarized monochromatic light.

The characteristic in-flow patterns of windows in whole pipe cross-section shavings were observed under the transmitted white light (Figure 6a), monochromatic light (Figure 6b), cross-polarized white (Figure 6c), and monochromatic (Figure 6d) lights. The windows were best observed with a monochromatic cross-polarized light, followed by cross-polarized white light. The shavings of sample 4 viewed under a cross-polarized monochromatic light are shown in Figure 7. The shaving thickness varied from 50 µm to 300 µm, and a higher area fraction of windows (% windows) appeared in the thinner shavings, as shown. Furthermore, at shaving thicknesses above 150 µm, some of the features of the original windows observed at thinner shaving thicknesses disappeared in polarized light. Therefore, in terms of making whole pipe shavings from the pipe, a good visual observation of the windows, ease of handling, and testing shavings, a 100 µm thickness was found to be the most suitable for use.

**Figure 8:**
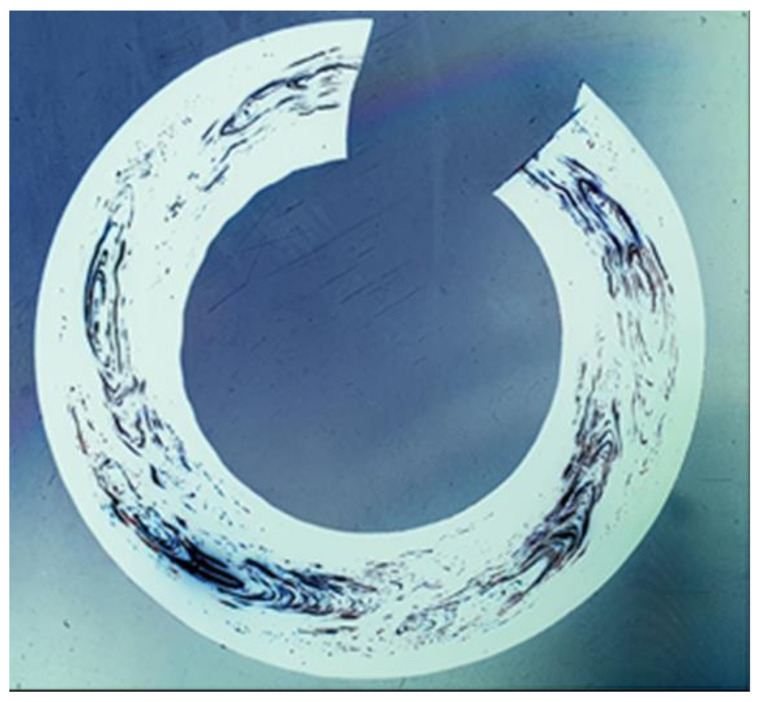
Color-inverted image of sample 4 (100-μm shaving in Figure 7).

Figure 8 shows a color-inverted image of a shaving. Although the observed window details were similar, using both original and color-inverted images can complement each other to improve the viewing of the details of the windows’ patterns.

**Figure 9:**
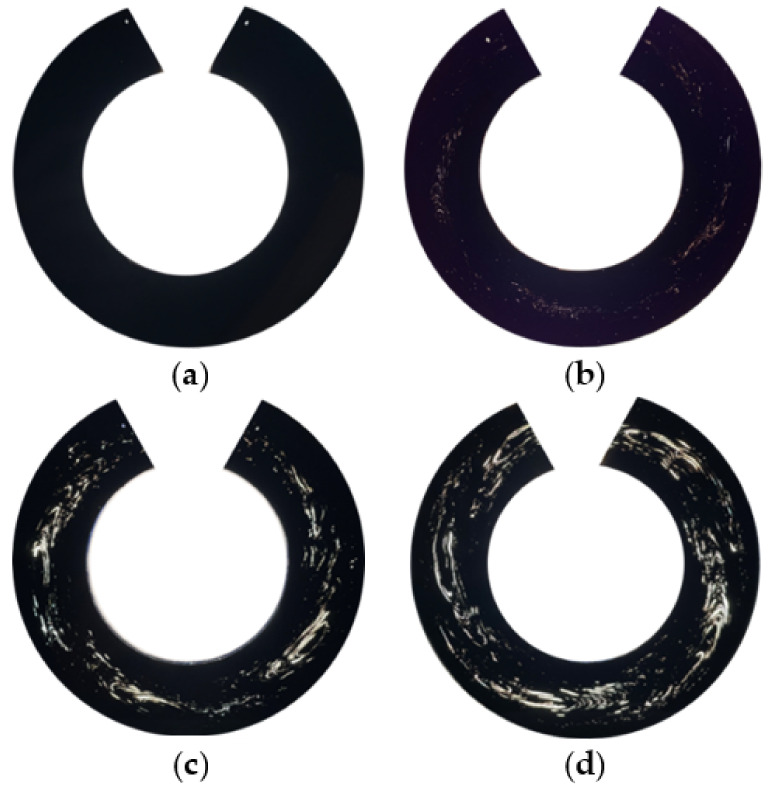
Whole pipe cross-section shavings under cross-polarized monochromatic light. (**a**) Sample 1, (**b**) sample 2, (**c**) sample 3, and (**d**) sample 4.

The 100 µm thick shavings of the whole pipe cross section for all pipe samples are shown in Figure 9. In sample 1, no windows appeared as it was made windows-free, whereas highly directional window swirls in the in-flow direction were evident in the shavings from samples 2 to 4. The intensity of the window swirls diminished with decreasing levels of windows in the shavings; they all appeared to be grouped into four distinct swirls’ patterns (Figure 9b–d). The formation of such characteristic swirls’ patterns of windows was due to using a four-channel spiral die during pipe extrusion in a single screw extruder.

**Figure 10:**
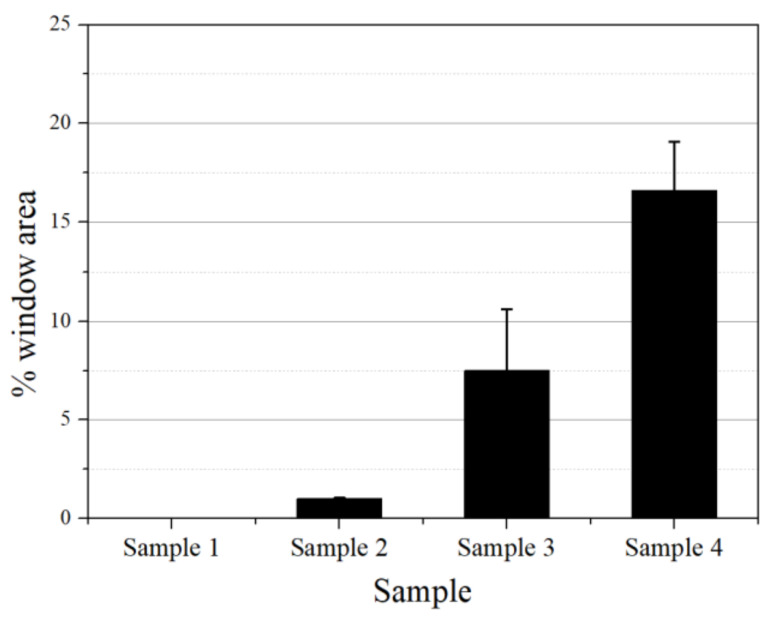
The % windows determined by the grayscale pixel count on 100 μm whole pipe cross-section shavings under monochromatic cross-polarized light.

The % windows of shavings determined using the monochromatic cross-polarized light are shown in Figure 10. The mean % windows were found to be 0%, 1.5%, 7.5%, and 16.5%, for samples 1, 2, 3, and 4, respectively.

**Figure 11:**
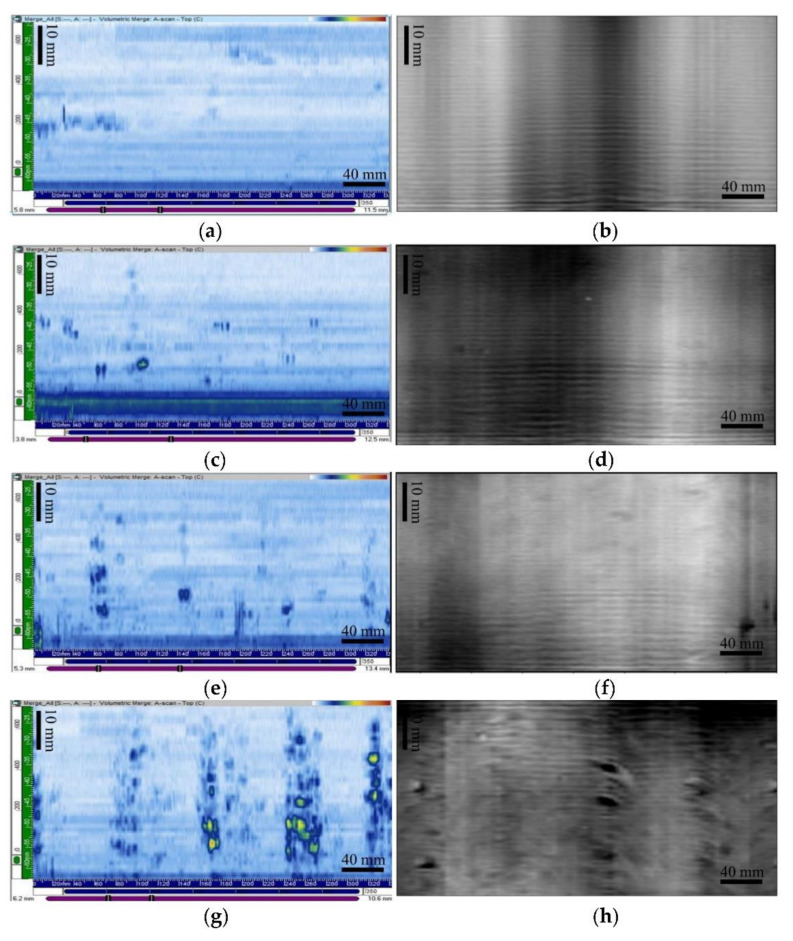
C-scan PAUT (**left**) and MW (**right**) images. (**a**,**b**) Sample 1, (**c**,**d**) sample 2, (**e**,**f**) sample 3, and (**g**,**h**) sample 4. In both the PAUT and MWI images, the horizontal axis is the pipe circumference from 0 to 360 degrees, and the vertical axis is an axial length of approximately 50 mm.

Figure 11 shows the resulting C-scan images from the PAUT inspection for sample 1 (Figure 11a), sample 2 (Figure 11c), sample 3 (Figure 11e), and sample 4 (Figure 11g) pipes. The horizontal axis of the C-scan image represents the full pipe circumference, and the vertical axis represents the inspection area in the axial direction of the sectorial scan. It can be seen that the amplitude of the reflected ultrasonic signals varied according to the level of windows present; thus, its distribution could be determined. What appeared to be columns of particulate indications in samples 2 to 4 was correlated to the window swirl clusters observed in the shavings, as shown in Figure 9. No PAUT indication of windows was found in sample 1 (windows free, Figure 9a and Figure 11a).

Similarly, window indications in the form of particulate column images were also displayed with MWI NDT (Figure 11b,d,f,h). The window indications increased going from samples 2 to 4. As with PAUT, no MWI indications of the window were found in sample 1 (Figure 11b). Both the PAUT and MWI indications showed windows clusters in the direction of the pipe axis. These NDT images can be further sectioned into known areas, and the number and size of windows present in the image can be counted and binned by their relative sizes. This would allow the level of windows to be quantitatively compared and the ranking determined by actual numbers rather than a simple visual comparison. Both approaches have merits and could be reviewed further in the future as the need arises. Therefore, PAUT and MWI are viable NDT methods for windows detection in polyethylene pipes.

**Figure 12:**
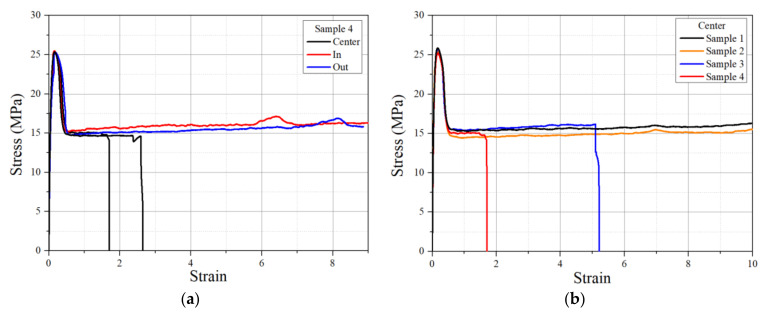
Stress–strain curves of (**a**) 3.5 mm-thick specimens taken from the inner, mid, and outer walls of sample 4 and (**b**) specimens from the center wall of all pipe samples.

Engineering tensile stress–strain curves from 3.5 mm thick specimens are illustrated in Figure 12. Tensile yield stress of about 25 MPa was indicated for all specimens independent of the position they were removed from. Additionally, as expected, the stress–strain behavior of all specimens from the inner and outer layers was not affected as these were from the pipe thickness free of windows (Figure 12a and Figure 3). However, for specimens taken from the center pipe wall, fracture strains decreased in samples 3 and 4, and no decrease was observed in samples 1 and 2 (Figure 12b). The stress–strain behavior of 3.5 mm thick specimens confirmed the visual observations of the windows being positioned at the inner portion of the pipe wall for pipe samples 3 and 4. This was due to the higher shear stresses for CB mixing produced at the inner and outer walls of the pipe during pipe extrusion. On the other hand, the absence of fracture strain change for specimens from samples 1 and 2 indicated some minimum % windows are needed to cause a fracture strain decrease.

**Figure 13:**
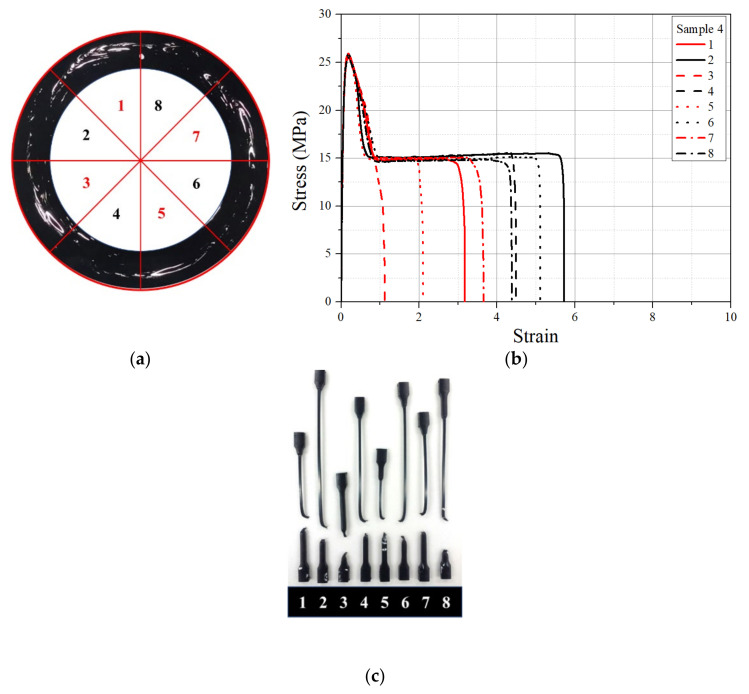
The 12 mm thick tensile specimens from pipe sample 4. (**a**) Eight sector locations, (**b**) tensile stress–strain curve of each specimen, and (**c**) tested specimen.

**Figure 14:**
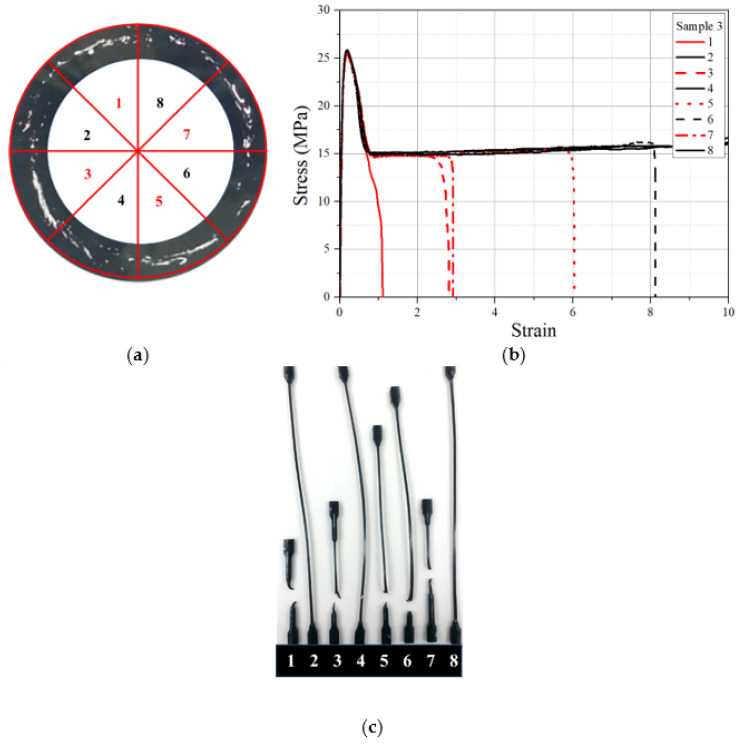
The 12 mm thick tensile specimens from pipe sample 3. (**a**) The eight sector locations, (**b**) tensile stress–strain curve of each specimen, and (**c**) tested specimen.

**Figure 15:**
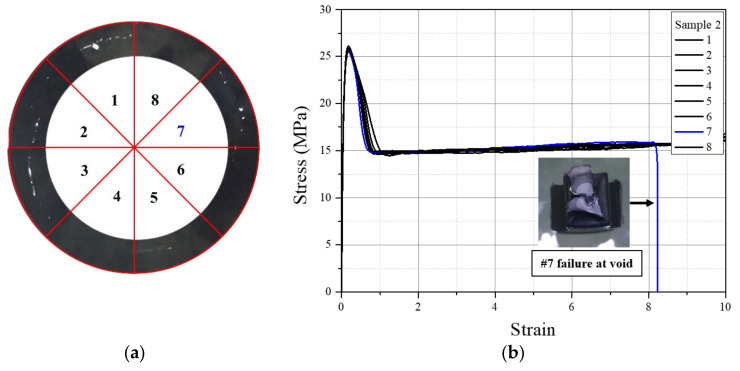
The 12 mm thick tensile specimens from pipe sample 2. (**a**) The eight sector locations, and (**b**) tensile stress–strain curve of each specimen.

In order to see the effect of % windows on the stress–strain behavior of the pipe samples, the tensile results from the 12 mm thick specimens were considered. The stress–strain behaviors of 12 mm thick tensile specimens made at eight locations in the cross section with known % windows are shown in Figure 13, Figure 14 and Figure 15 for pipe samples 4, 3, and 2, respectively. In pipe sample 4, the fracture strain was drastically reduced for all specimens, as seen from the stress–strain graph and the actual failed samples. Tensile specimens 1, 3, 5, and 7 from pipe sample 4 were obtained from high-intensity window swirl locations (Figure 13a), and all showed higher fracture strain reduction (Figure 13b,c). Additionally, as expected, specimens 2, 4, 6, and 8 exhibited less reduction (Figure 13b,c), as they represented specimens from the lower % windows’ portion of the pipe cross section (Figure 13a). A fracture strain reduction pattern was also observed in tensile specimens 1, 3, 5, and 7 and 2, 4, 6, and 8 from pipe sample 3, as shown in Figure 14. For specimens from pipe sample 2, as with pipe sample 1 (windows free), no position dependence of the fracture strain was found, as seen in Figure 15. A single specimen from pipe sample 2 had a premature failure before reaching the maximum strain value, and this was due to the presence of voids, as shown in Figure 15b.

**Figure 16:**
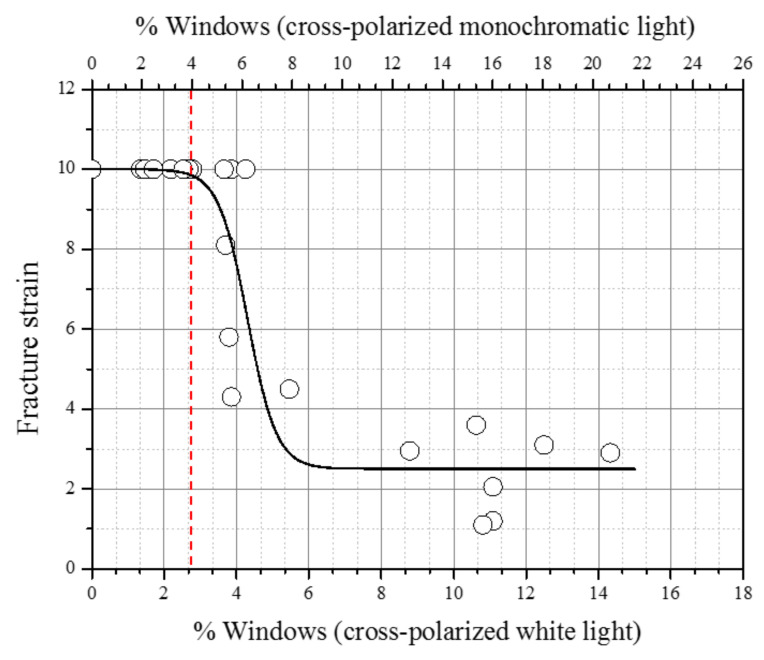
Fracture strain versus % windows of specimens from all sectors.

Figure 16 illustrates the correlation between the % windows determined at each of the eight sectors and the corresponding specimen fracture strains for all pipe samples. The fracture strain was seen to undergo no decrease until about 2.5% windows; then, a rapid decrease to about 250% limiting strain value occurred. From this behavior, one can estimate the limiting % windows at which the integrity of the tensile specimen can be maintained. It is noted that different lighting conditions used to determine the % windows can provide different results. For example, the limiting fracture strain of 2.5% determined with a white cross-polarized light became about 4.0% when a monochromatic cross-polarized light was used (Figure 16). Hence, the type of lighting and the shaving thickness used need to be mentioned when determining the % windows present.

**Figure 17:**
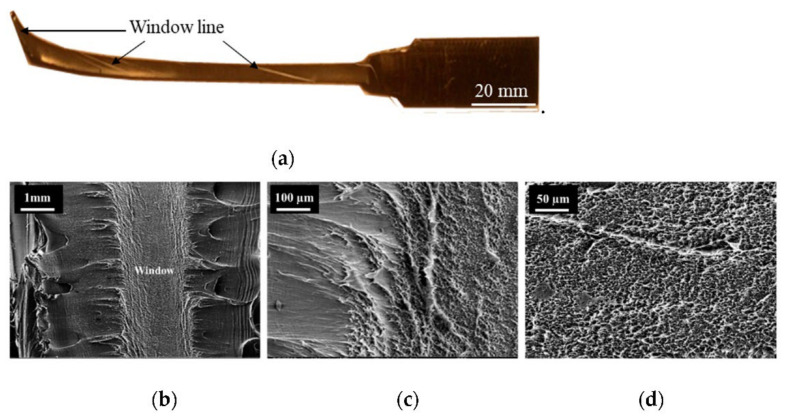
(**a**) Twisted window line along the cold drawn length of the tensile specimen and fracture surface morphology at (**b**) ×40, (**c**) ×200, and (**d**) ×500.

Figure 17 shows the fracture surface of a tensile specimen taken from pipe sample 4. In the cold drawn region of the tensile tested specimen (Figure 17a), windows in the form of a twisted line can be observed along the length of the specimen up to the point of fracture (see the arrows). The fracture surface examined is shown in Figure 17b–d. At lower magnifications (×40 and ×200), the fracture surface was seen to contain a region of flatter fracture sided by tearing from gross yielding. A further examination of the flat fracture at higher magnification (×500) showed a short, fibrillated surface, indicating micro-ductility in the windows. In contrast, the yielding tear adjacent to the fibrillated surface indicated shear yielding failure in the black compound area.

Therefore, it was clear that the windows’ fracture produced a fibrillated fracture surface, whereas the black compound failed via a gross yielding tear. Hence, the cause for the reduction in the global fracture strain can be attributed to the presence of windows.

**Figure 18:**
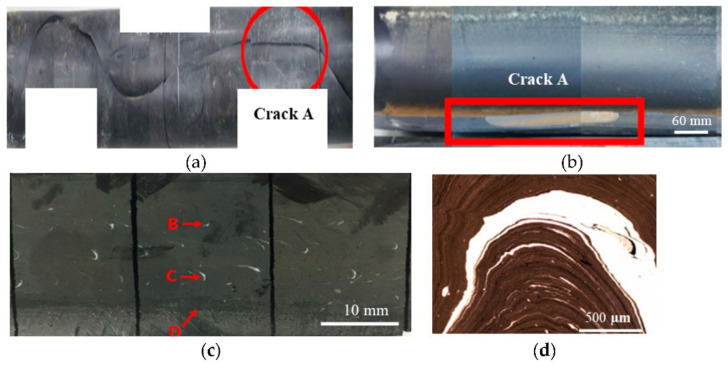
(**a**) Rapid crack propagation failure, (**b**) fracture surface at “crack A” indicating slow crack growth, (**c**) “crack A” fracture surface containing windows, and (**d**) windows swirls.

**Figure 19:**
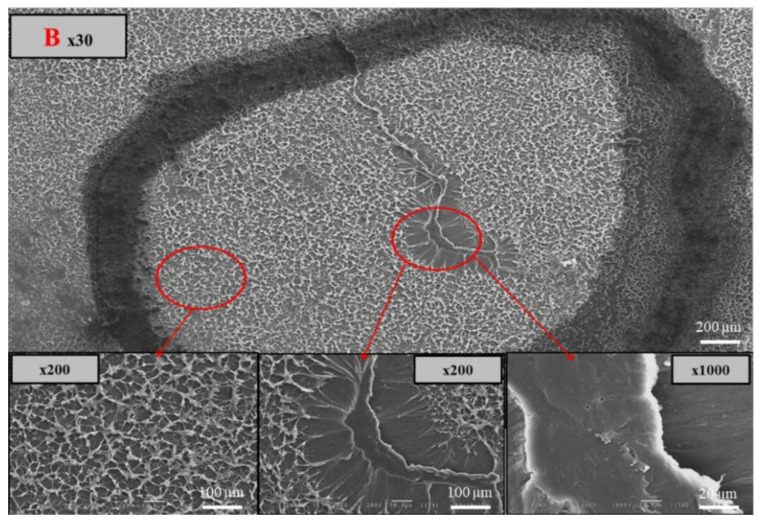
SEM photomicrographs of the fracture surface of the window and black compound regions (region B in Figure 18c).

In support of this, Figure 18a shows a rapid crack propagation (RCP) failed 315 SDR 11 black HDPE water pipe and the slow crack growth preceding RCP failure (Figure 18b). The windows are clearly visible on the slow crack surface toward the mid-pipe wall (Figure 18c). They were further confirmed by the white swirls observed in the 15 µm thick film microtomed from the windowed area (Figure 18d). Figure 19 shows photomicrographs of the windowed area B (Figure 18c), seen under a scanning electron microscope (SEM). The windows exhibited a flat, brittle fracture surface. In contrast, the typical micro-ductility fibril morphology of the fracture surface was apparent in the black compound area, indicating that slow crack growth had occurred. Hence, the flat, brittle fracture of the local windows is thought to provide a path for premature failure.

It is noted that the window fractures exhibited different fracture morphology comparing the tensile tested specimen (Figure 17) to the pipe specimen that underwent slow crack growth before the rapid crack propagation failure (Figure 18). The fracture morphology difference was likely due partly to the different loading and specimen geometries between the two cases. Nevertheless, these two examples clearly indicated that the presence of windows was a contributing factor leading to the premature failure of PE pipes.

**Figure 20:**
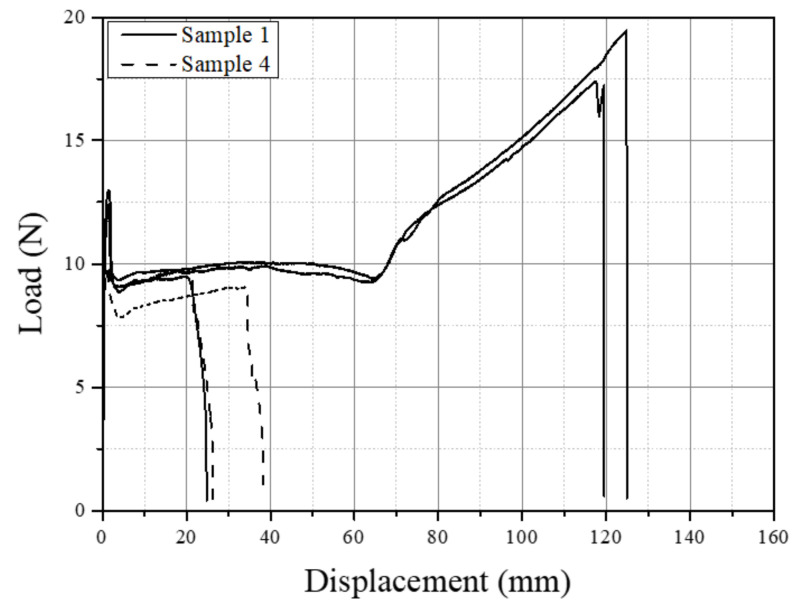
Load–displacement curves of tensile-tested 100 µm thick shaving from samples 1 and 4.

**Figure 21:**
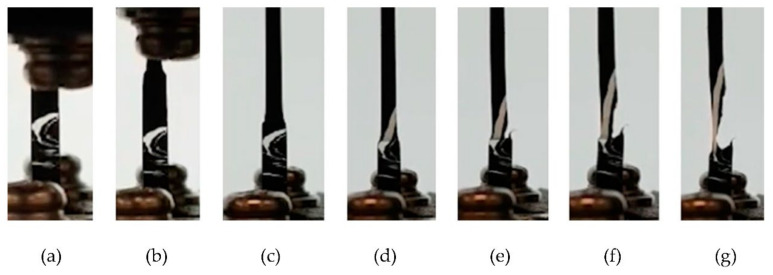
Frame-by-frame photographs showing the progression of windows’ deformation to tear fracture.

To further understand how fractures initiate in a static tensile test with windows present, a tensile test was performed on micro-tensile specimens produced from the shavings of samples 1 (windows free) and 4 (high windows). The micro-tensile specimens from sample 4 were taken at four locations along the pipe circumference where intense window swirls were present, as shown in Figure 4. Tensile test results are exhibited in Figure 20. Distinct regions of initial displacement to tensile yield load (onset necking), neck formation, cold drawing, and orientation hardening prior to fracture for sample 1 can be observed. For sample 4, all tensile specimens failed during the early part of cold drawing and did not reach orientation hardening displacement. This is consistent with what was observed for the thicker tensile specimens made from samples 1 and 4 (Figure 13). The fracture mechanism can be inferred by observing the frame-by-frame photos of the tensile test, as given in Figure 21. The window swirl underwent uniform deformation up to yield (a); the neck formed and the cold drawing began (b); the cold drawing reached the window swirl (c); the cold drawing included windows and continued without any disturbance to the main window (d); further cold drawing caused a tear crack to initiate at the secondary window swirl below (e); the tear propagated (f) until a final failure occurred (g). The tear crack seemed to initiate at the boundary between the secondary window swirl and the black compound region between the main and secondary window swirls (e). Deveci and coworkers [23] also reported the boundary crack initiation in tensile testing of a 15 µm thick microtomed film containing windows. They attributed this to the higher CB concentration at the window–black compound boundary due to the interfacial free energy difference.

**Figure 22:**
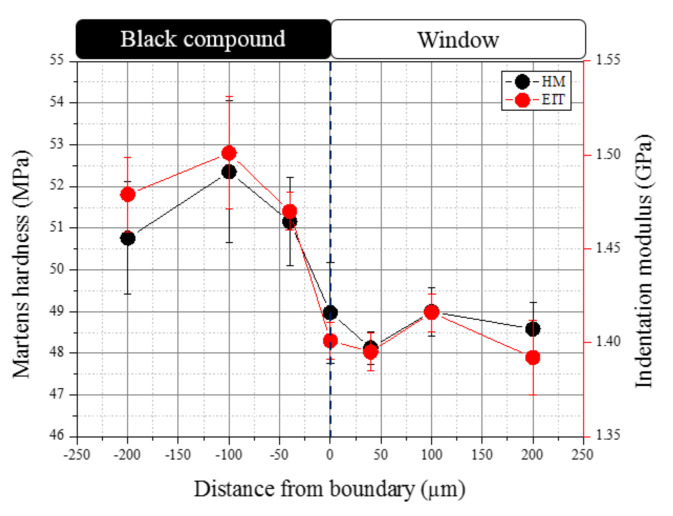
Variation of HM and EIT with distance from the windows–black compound boundary.

To possibly confirm the effect of CB concentration on tear initiation, micro-indentations were made at various locations at specific distances away from the window–black compound boundary, as shown in Figure 5. The micro-indentation results are shown in Figure 22. The Martens hardness (HM) and indentation modulus (EIT) both increased, moving into the black compound area, reaching a maximum value at 100 μm from the boundary, and then began to slightly decrease to a lower value deeper in the black area (200 μm). On the other hand, the lower values were approximately maintained into the windows area. This confirmed that the presence of CB particles increased the hardness and modules of PE [31]. However, with only about a 7% higher indentation modulus of the black compound (1.5 GPa) compared to the window (1.4 GPa), the shear stress developed at the boundary was unlikely to be high enough to cause ductile tear initiation at the boundary. Further studies are needed to determine the mechanism of tear initiation at the window–black compound boundary.

## 4. Conclusions

In this work, polyethylene (PE) pipes with varying amounts of windows were investigated for their characteristic window patterns, and the area fraction of windows (% windows) was quantified using visual and non-destructive examinations. Additionally, a tensile test was employed to determine the limiting % windows for the onset of rapid fracture strain degradation. In addition, an attempt was made to ascertain the mechanism of fracture initiation in the presence of windows by using a micro-tensile test and micro-indentation on thin shavings from the pipes. The main results are the following.

(1) A turning method of producing 110 SDR 5 whole PE pipe cross-section shavings of thickness as small as 30 μm was developed, and we demonstrated that a 100 μm thick shaving is best for performing visual observation and micro-tensile tests.
(2) Visual observation of the windows and their swirl patterns in a whole pipe cross section was determined to be best offered by using a cross-polarized monochromatic light. In addition, the windows’ locations and swirl patterns provided CB mixing details in a single screw extrusion.
(3) The % windows in a whole pipe cross section and sectorial sections using images from the visual windows’ observations were measured, and 0%, 1.5%, 7.5%, and 16.5% were determined for windows free (sample 1), low (sample 2), medium (sample 3), and high (sample 4) levels of window-containing pipes, respectively.
(4) Tensile test performed on specimens from pipes containing various % windows showed a limiting % windows value of 4.0% (cross-polarized monochromatic light), above which caused a rapid decrease in fracture strain as much as four times the window free specimen. A lower % windows value of 2.5% was determined using cross-polarized white light, thus indicating the % window determination is dependent on the lighting and shaving thickness employed.
(5) Windows indications and similar patterns were also observed with the phased array ultrasonic (PAUT) and microwave imaging (MWI) non-destructive examination methods. They are both confirmed to be viable methods for detecting windows directly on pipes.
(6) The fracture surface morphology of the tensile specimens showed that the micro-ductility fibrils were characteristic of the windows’ fracture, while the black compound exhibited a yielding failure, confirming that windows are a degrading factor in the structural integrity of the PE pipe.
(7) The micro-tensile test showed failure initiation at the windows–black PE compound boundary at the post-necking strain. However, since the micro-indentation modulus increased only about 7% in the black compound compared to the windows, a definite conclusion cannot be drawn regarding the cause for the boundary area fracture initiation.

The results of this study have further indicated that a better compounding method (e.g., Sample 1) would be preferred to avoid possible issues affecting the structural integrity of the PE pipes. In addition, since PAUT and MWI non-destructive techniques are confirmed as viable for the direct inspection of pipes containing windows, procedures should be developed for ranking between pipes.

## Figures and Tables

**Table 1:** Identification and characteristics of pipe samples.

Materials	Level of Window	Extrusion Throughput	CB Content	MFR5	MFR21	Density
		kg/h	wt%	(g/10 min)	(g/10 min)	(g/cm^3^)
Pre-compound			2.11	0.27	8.76	0.96
NPC			NA	0.27	8.77	0.95
Sample 1	Windows free	115	2.11	0.25	8.07	0.9603
Sample 2	Low	70	2.37	0.28	8.26	0.9609
Sample 3	Medium	95	2.40	0.26	7.99	0.9606
Sample 4	High	115	2.26	0.27	8.09	0.9607

## Data Availability

The data that supports the findings of this study are available within the article.
